# Electrospun fibrillary scaffold for electrochemical cell biomarkers detection

**DOI:** 10.1007/s00604-024-06523-w

**Published:** 2024-06-29

**Authors:** Mihaela Beregoi, Daniela Oprea, Mihaela Cristina Bunea, Monica Enculescu, Teodor Adrian Enache

**Affiliations:** 1 Functional Nanostructures Laboratory, National Institute of Materials Physics, Atomistilor Str. 405A, 077125 Magurele, Romania; 2 Faculty of Physics, University of Bucharest, Atomistilor Str. 405, 077125 Magurele, Romania

**Keywords:** Electrospinning, Nanofibers, Scaffold, Melanin, B16 cell line, Electrochemical detection

## Abstract

A novel scaffold for in situ electrochemical detection of cell biomarkers was developed using electrospun nanofibers and commercial adhesive polymeric membranes. The electrochemical sensing of cell biomarkers requires the cultivation of the cells on/near the (bio)sensor surface in a manner to preserve an appropriate electroactive available surface and to avoid the surface passivation and sensor damage. This can be achieved by employing biocompatible nanofiber meshes that allow the cells to have a normal behavior and do not alter the electrochemical detection. For a better mechanical stability and ease of handling, nylon 6/6 nanofibers were collected on commercial polymeric membranes, at an optimal fiber density, obtaining a double-layered platform. To demonstrate the functionality of the fabricated scaffold, the screening of cellular stress has been achieved integrating melanoma B16-F10 cells and the (bio)sensor components on the transducer whereas the melanin exocytosis was successfully quantified using a commercial electrode. Either directly on the surface of the (bio)sensor or spatially detached from it, the integration of cell cultures in biosensing platforms based on electrospun nanofibers represents a powerful bioanalytical tool able to provide real-time information about the biomarker release, enzyme activity or inhibition, and monitoring of various cellular events.

**Graphical Abstract:**

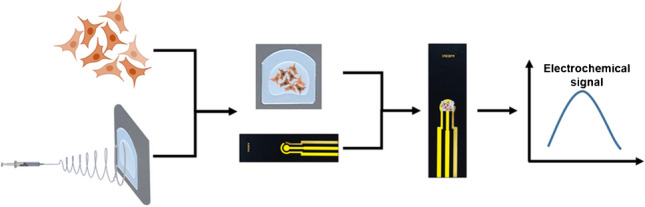

**Supplementary Information:**

The online version contains supplementary material available at 10.1007/s00604-024-06523-w.

## Introduction

The most amazing biological structure is the living cell, a dynamic machine that integrates a wide variety of biochemical structures, continuously adapting and responding to the local environment. The cellular systems produce and transform signaling biomarker molecules to inter-communicate, and their integration in biosensing devices became an important tool to understand the underlying mechanisms of different diseases, as well as to perfect timely disease diagnosis and personalized therapeutic approaches [1]. Various techniques are commonly employed for detection and quantification of biomarkers generated by the cells in culture medium, the most used ones being spectroscopic techniques [2, 3], imaging methods [4], and microfluidics-assisted detection [5, 6]. Other techniques involved in biomolecules detection from cell culture are RT-PCR [7], ELISA [8], and HPLC [9] methods that employ expensive equipment and reagents with complex protocols. Some molecules were in vitro identified by using the mentioned techniques along with biomarkers such as fibroblast activation proteins, micro-RNA, proteome and phosphoproteome, and DNA oxidation biomarker [10–12].

An efficient determination and quantification of small molecules such as lactate, glucose, or reactive oxygen species [13–15] were consistently reported by integrating electrochemical (bio)sensor for on-line monitoring. Although the use of the electrochemical detection presents several conveniences such as easily miniaturization, high sensitivity and low limits of detection, issues similar with that presented in the “Issues in electrochemical detection” section could be encountered.

To avoid the mentioned problems, we propose within this study a novel approach involving a polymeric framework designed to support the growth and proliferation of cells, offering a suitable environment for integration onto a (bio)sensor surface. For the construction of this scaffold, we employed a nylon 6/6 network comprised of nanoscale fibers, which were electrospun onto a commercially available adhesive polymeric membrane. Electrospinning is a straightforward fiber preparation technique that uses an electric force to stretch and to elongate a polymer droplet in order to obtain a fiber [16] and has been used to design electrochemical (bio)sensors in order to ensure optimal support for cell growth, enhancing device characteristics allowing for customization of properties to align with cell morphology, composition, and mechanical stability [17]. In order to prove its functionality, this platform was used for detection of melanin produced by the melanoma B16 cells under UV stimulation. The choice of B16-F10 line and melanin as biomarker was a consequence of the fact that although the melanin expression in cellular medium it is well known, its propensity for aggregation represents a real challenge. As it has already be mentioned, the detection of melanin released by stressed melanocytes in culture medium has been already reported in scientific literature by using spectroscopic techniques and to our knowledge, the in vitro electrochemical detection has not been reached so far, considering the low volumes of produced melanin and the limitations presented in the “Issues in electrochemical detection” section.

Over time, various methods have been employed for melanin detection, starting with the utilization of the HPLC method in 1983 which has proven to be a valuable tool for exploring melanin composition, isolating, characterizing, and quantifying its individual components. While this analytical method offers several advantages, including accurate quantification and the capacity to isolate and characterize melanin components, it also presents certain drawbacks, such as the potentially damaging effects of the degradation process and the intricacies associated with sample preparation and analysis [18]. Moreover, spectrophotometric quantification of melanin is another method of quantification the amount of melanin in a sample by measuring the absorbance of light at certain wavelengths. It is a relatively simple and inexpensive method that can be used in a variety of samples, including hair, skin, and blood which can also estimate the relative ratio of eumelanin to pheomelanin [19]. However, even spectrophotometric quantification of melanin has some disadvantages. One disadvantage is that background absorbance from tissue components, such as proteins, can interfere with the measurements and overvalues of melanin content may result. Another disadvantage is that this technique cannot differentiate between the cells that can undergo melanogenesis and those who don't. On the other hand, fluorescent quantification of melanin is a nondestructive and more sensitive method than spectrophotometric quantification but is not as sensitive as HPLC, and it can be used in a variety of samples, but extensive attention must be directed to the possibility of other fluorescent molecules interference in the sample [20].

Thus, the electrospun fibrillary scaffolds aim to become powerful supports for cell growth and/or acting even as traducers in order to develop (bio)sensors with high sensitivity and selectivity for almost any kind of cell culture.

## Experimental section

### Materials and reagents

For the electrospinning process, poly(hexamethylene adipamide) (nylon 6/6, pellets, Sigma-Aldrich, St. St. Louis, MO, USA; CAS nr. 32,131–17-2) and formic acid (ACS reagent, ≥ 88.0%, Sigma-Aldrich, St. St. Louis, MO, USA; CAS nr. 64–18-6) were used as received. Commercial adhesive polymeric membranes (membranes for screen-printed electrodes, Methrom DropSens) were used as support for the electrospun nanofibers. Poly-L-lysine solution (0.01%, sterile-filtered, BioReagent, suitable for cell culture, Sigma, ST. St. Louis, MO, USA; CAS nr. 25,988–63-0) was used to improve the cell adhesion.

The reagents used for biological assays were as follows: melanoma cell line B16-F10 (ATCC, Manassas, Virginia, USA; CAT nr. CRL-6475™) and fibroblast cell line L929 (ATCC, Manassas, Virginia, USA; CAT nr. CCL-1), Dulbecco’s modified Eagle medium (DMEM, ATCC, Manassas, Virginia, USA; CAT nr. 30–2002™), trypsin (2.5%, no phenol red, ThermoFisher Scientific, Waltham, Massachusetts, USA; CAT nr. 15,090,046), phosphate buffer saline (PBS (10X), pH 7.4, ThermoFisher Scientific, Waltham, Massachusetts, USA; CAT nr. 70,011,044), fetal bovine serum (non-USA origin, sterile-filtered, suitable for cell culture, Sigma-Aldrich, St. Louis, MO, USA; CAS nr. 1,943,609–65-1), penicillin–streptomycin (10,000 U/mL, ThermoFisher Scientific, Waltham, Massachusetts, USA; CAT nr. 15,140,122), formaldehyde (ACS reagent, 37 wt. % in H_2_O, contains 10–15% methanol as stabilizer (to prevent polymerization), Sigma-Aldrich, St. Louis, MO, USA; CAS nr. 50–00-0), glutaraldehyde solution (50 wt. % in H_2_O, Sigma-Aldrich, St. Louis, MO, USA; CAS nr. 111–30-8), CellTiter 96®AQueous One Solution Cell Proliferation Assay (MTS, Promega Corp., Wisconsin, SUA; CAT nr. G3582), and CyQUANT™ Cell Proliferation Assay (for cells in culture, ThermoFisher Scientific, Waltham, Massachusetts, USA; CAT nr. C7026). Osmium tetroxide (4 wt. % in H_2_O, Sigma-Aldrich, St. Louis, MO, USA; CAS nr. 20,816–12-0) is commonly utilized as a post-fixative and staining agent to enhance the contrast of the samples and it was used for morphological analysis. For fluorescence microscopy visualization, 4′,6-diamidino-2-phenylindole (DAPI, ThermoFisher Scientific, Waltham, Massachusetts, USA; CAT nr. D1306), acridine orange (10 mg/mL in water, ThermoFisher Scientific, Waltham, Massachusetts, USA; CAT nr. A3568), and Phalloidin-iFluor™ 647 (ThermoFisher Scientific, Waltham, Massachusetts, USA; CAT nr. 20,555) were used as dyes and FluorSave™ Reagent (Merck Millipore, Massachusetts, USA; CAT nr. 345,789) for preserving the fluorescence.

The fabricated sensing fibrillary platform was tested for in vitro melanin detection under irradiation by using commercial screen-printed carbon electrodes (with working electrode carbon (4-mm diameter), auxiliary electrode platinum, and reference electrode silver, Methrom DropSens; Ref. 150). Synthetic melanin (BioReagent, suitable for cell culture, Sigma-Aldrich, St. St. Louis, MO, USA; CAS nr. 8049–97-6) was used for standardization.

All solutions were prepared by utilizing purified water from a Millipore Milli-Q system with conductivity ≤ 0.1 µS cm^−1^.

### Instrumentation

Nanosized fibers were prepared employing a New Era Pump System to feed the nylon 6/6 solution through a blunt needle, a Spellman SL300 power supply with an output of 50 kV and an aluminum plate grounded collector with the commercial polymeric membranes attached on it. The electrospinning equipment was placed into a chamber that allows a precise control of temperature and humidity.

The fluorescence microscopy images were obtained using a Leica DM6B upright fluorescence microscope (Leica Microsystems CMS GmbH, Wetzlar, Germany) equipped with a Leica CTR6 LED and a Leica EL6000 external light source for fluorescence excitation. The samples were analyzed utilizing a 40X objective (0.65 NA, 0.36 mm WD, and correction ring) from Leica, the appropriate filter cube (excitation filter 325–375 nm, dichroic mirror 400 nm and emission filter 435–485 nm for nuclei, excitation filter 460–500 nm, dichroic mirror 505 nm and emission filter 512–542 nm for nuclei and excitation filter 590–650 nm, dichroic mirror 660 nm and emission filter 662–738 nm for actin filaments) and the 4.2 MP sCMOS Leica DFC9000 monochrome fluorescence camera. The fluorescence microscopy images were taken before cell staining by using the following procedure: the cell/scaffold complexes were incubated for 10 min at room temperature in DAPI dye for highlighting the cellular nuclei and Phalloidin-iFluor™ 647 dye for evidencing the cytoskeletal structures; after this, the samples were rinsed with PBS for removing the dye traces and transferred on microscopic slides using FluorSave™ Reagent.

The morphological analysis was carried out by employing a Zeiss SEM 500 Gemini Field Emission Scanning Electron Microscope (SEM) equipped with energy dispersive X-ray analysis Quantax XFlash detector 610 M (Bruker, Billerica, Massachusetts, USA). SEM images of seeded samples were recorded after a post-fixation of cells with 0.1% osmium tetroxide solution for 15 min at room temperature. After drying the samples, any residual traces of osmium were eliminated through three successive PBS rinses.

Electrochemical measurements were conducted using a potentiostat/galvanostat (Ivium Technologies, Eindhoven, The Netherlands) running an IviumSoft 4.977 (Ivium Technologies, The Netherlands) and a three-electrode system including the prepared adhesive scaffolds implanted with melanoma B16 cells that were attached on the screen-printed carbon electrodes.

### Methods

#### Preparation of the electrochemical detection scaffold

**Scheme 1:**
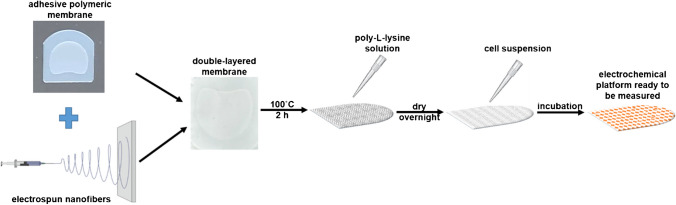
Preparation steps of the electrospun fibrillary scaffolds for cell culture support

Fibrillary scaffolds were obtained by combining two important elements (according to Scheme 1), namely, electrospun nanofibers and commercial adhesive polymeric membranes, removing in this way the shortcomings of using each individual element (e.g., poor mechanical resistance of the free-standing nanofibers and weak adhesion of cells on the polymeric membranes). The nanofiber meshes were produced by electrospinning a 17% (w/v) nylon 6/6 solution which was obtained by dissolving the corresponding amount of monomer in formic acid. The electrospinning process was conducted by applying 25 kV, placing the collector 20 cm apart from the needle and feeding the solution with 0.1 mL/h. The collector consisted of a grounded aluminum plate with the adhesive polymeric membranes attached on it, in order to collect the nanofibers directly on the membranes. The working temperature ranged between 21 and 24 °C and the relative humidity was maintained between 35 and 40%. To achieve uniform fiber densities, the collection time was kept constant. Afterwards, the fibrillary platforms were thermally treated at 100 °C for 2 h in the oven for removing the solvent traces from the electrospinning process and improving the adhesion of the nanofibers on the polymeric membranes. For enhancing the cell adhesion, 70 μL from 0.01% poly-L-lysine solution was placed on top of the nanofibers and left overnight to dry. It is worth to mention that after this step, the sterilization can occur and the platforms can be used for electrochemically analyzing almost any kind of cell culture either using a commercial electrode (such as DropSens screen-printed carbon electrodes) or metalizing the nanofibers, acting as a (bio)sensor traducer.

This approach removes difficulties such as (bio)sensor active surface passivation or damage, necessity of using high volumes of biomarkers or altering the cell culture viability or metabolism during measurements.

#### Cell cultivation and fixation

B16-F10 melanoma and fibroblast L929 cells were grown in Dulbecco's Modified Eagle Medium (DMEM) culture medium supplemented with 4.5 g/L glucose, 2 mM/L glutamine, 10% fetal bovine serum and penicillin (100 U/mL), and streptomycin (100 μg/mL), under controlled conditions (over 90% humidity, 5% CO_2_ and 37 C). Sub-cultivation was done in cell culture flasks (T-25), and when cells reached an optimum confluence ~ 80%, the cells were detached using trypsin solution of 0.25% concentration, counted and seeded on the designed fibrillary platform at a density of 14,000 cells/sample. The cells were analyzed after 24 h of incubation. The same procedure was performed for a control sample, namely, polystyrene plates.

To provide aseptic conditions for cells, the scaffolds were immersed for 6 h in ethanol 70%, kept in Millipore-Milli-Q purified water for 3 days to make sure all traces of ethanol were eliminated and kept for 30 min under UV light irradiation before being used.

For fixation, the electrospun fibrillary scaffolds on which the cells were seeded were immersed and incubated in 4% paraformaldehyde solution for 15 min at room temperature. After the incubation period, the paraformaldehyde trances were carefully removed from the samples by three successive PBS rinses.

#### Melanin detection

The functionality of the cell biomarker detection scaffold was demonstrated by identifying the melanin generated by the melanoma B16-F10 cells under irradiation. The first step consisted in cells seeding on the samples by using the above protocol, and after proliferation, the ensemble cells fixed on double-layer membrane was transferred onto the surface of a screen-printed carbon electrode. The next step involved the irradiation of the cell-seeded sensors with a 395-nm beam laser for different periods of time (5, 15, and 30 min). An intermediary step implied conditioning of the SPC electrode in diluted solutions of H_2_SO_4_, followed by PB pH = 7.4 by cyclic voltammetry between the potential limits *E*_*i*_ = 0.0 V, *E*_max_ =  + 1.4 V, *E*_min_ =  − 1.0 V, and *E*_*f*_ = 0.0 V. At the end, after each irradiation period a cyclic voltammogram was recorded between the potential limits used for conditioning the SPC electrodes. To quantify the melanin produced by cells during irradiation, various concentrations of melanin (20, 60, and 100 µM) were introduced and measured.

Electrodes reproducibility was assured based on several tests. In a previous study [21], after conditioning the electrodes, most of them exhibited similar behaviors, showing the same sensitivity and consistency in peak currents indicating that the activation procedure is effective in standardizing the electrode surfaces, leading to reproducible and reliable results in electrochemical experiments. Therefore, after applying the optimal activation procedure, the electrodes are achieving an uniform level of performance, which is essential for the validity and reproducibility of the results obtained in subsequent experiments. In this case, prior to attachment of the scaffold containing cells on the electrode surface, for each electrode utilized, an electrochemical treatment was used, and only the electrodes that presented the same baseline were employed on further. Also, each scaffold was seeded with the same number of cells and incubated at the same conditions.

## Results and discussion

### Issues in electrochemical detection

The primary benefit of employing sensors within cell cultures lies in their capacity to rapidly gauge cellular reactions to various stimuli. A straightforward approach involves immersing the (bio)sensor in the culture medium, thereby measuring cell responses subsequent to diverse stimuli. These responses manifest as the release of distinct molecules into the cellular milieu.

However, an issue arises from this approach, stemming from the low quantities of biological molecules that cells produce and discharge into the culture medium. Typically, these quantities are within the pico to nanomolar range [20]. Consequently, for analytical purposes, it becomes imperative to cultivate cells in close proximity to the sensor’s surface elevating this way the concentration of active molecules available for detection. The conventional method for fostering cell growth and proliferation at the (bio)sensor surface entails seeding cells onto the (bio)sensor [22, 23]. Subsequently, the (bio)sensor is incubated for a minimum of 24 h under controlled conditions, such as a temperature of 37 °C, 5% CO_2_, and over 90% humidity. Nonetheless, complications can arise from this process. Extended incubation can lead to the passivation of the (bio)sensor surface due to the adsorption of chemical species from the culture medium, rendering the (bio)sensor nonfunctional. Furthermore, during measurements, direct contact between cells and the (bio)sensor surface could potentially harm the cells due to the flow of charges between the electrodes. This interaction might also generate interfering molecular species as a response [24].

**Fig. 1:**
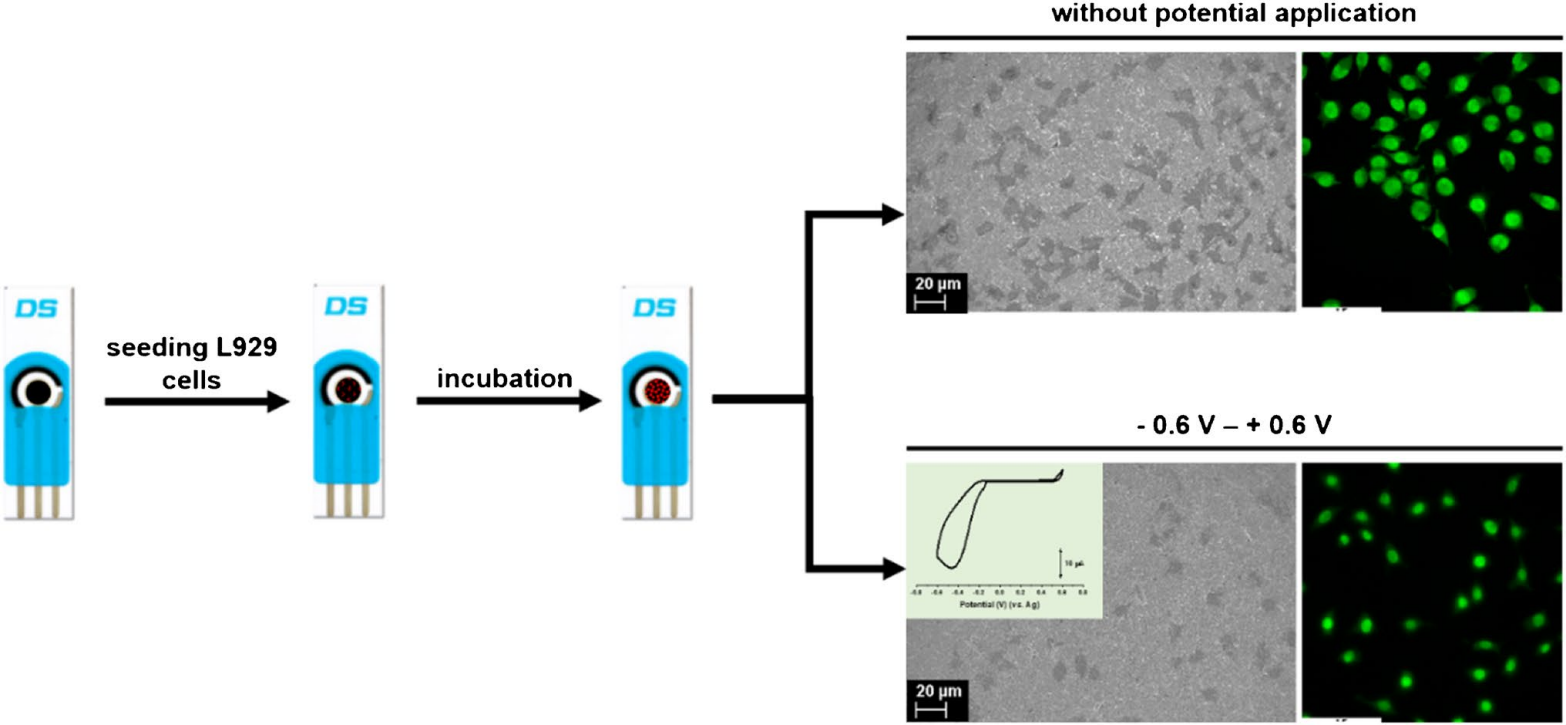
Schematic representation of issues that can appear during electrochemical cell culture investigations

An example related to the influence of charge flow on the cells culture at electrode surface, is given bellow. The fibroblasts L929 were seeded on DropSens carbon electrode. After 24 h of incubation, cyclic voltammogram, between potential limits *E*_*i*/*f*_ = 0 V, *E*_max_ =  + 0.6 V, and *E*_min_ =  − 0.6 V, was recorded for cell culture cultivated at the electrode surface (Fig. 1). Using acridine orange as nuclear stain, fluorescence images were recorded before and after electrode polarization and a decrease of cell population, associated with the flow charge between working and counter electrodes, was found for the samples investigated after cyclic voltammetry investigation (Fig. 1). Following cyclic voltammetry, it was observed that L929 fibroblasts cells manifested a change in their morphology resulting in a rounded fibrocystic cell structure, specific for inactive fibroblasts, with a noticeable size reduction of nuclei.

**Fig. 2:**
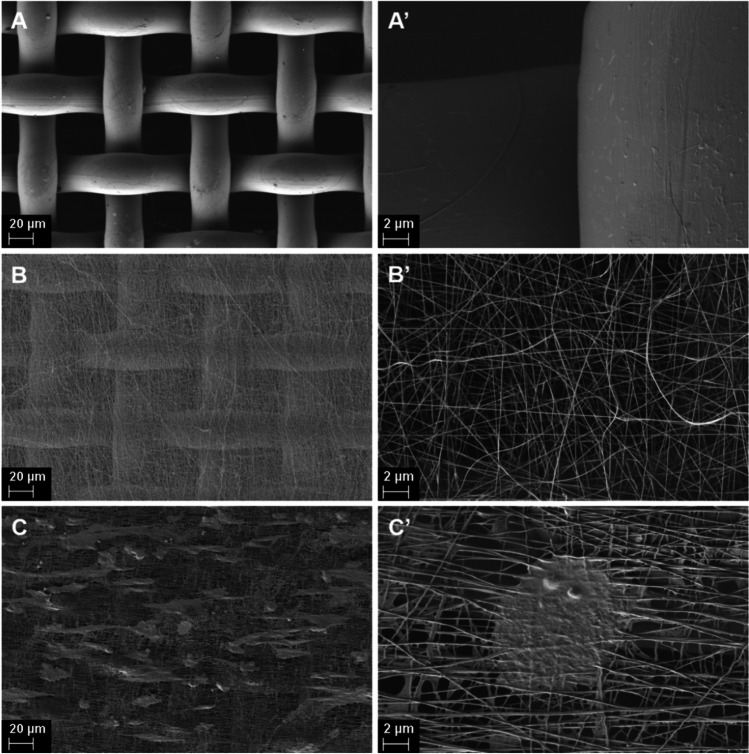
SEM images of the **A, A’** the commercial adhesive polymeric membrane and the electrospun fibrillary platform **B, B’** without and **C, C’** with B16 melanoma cells

**Fig. 3:**
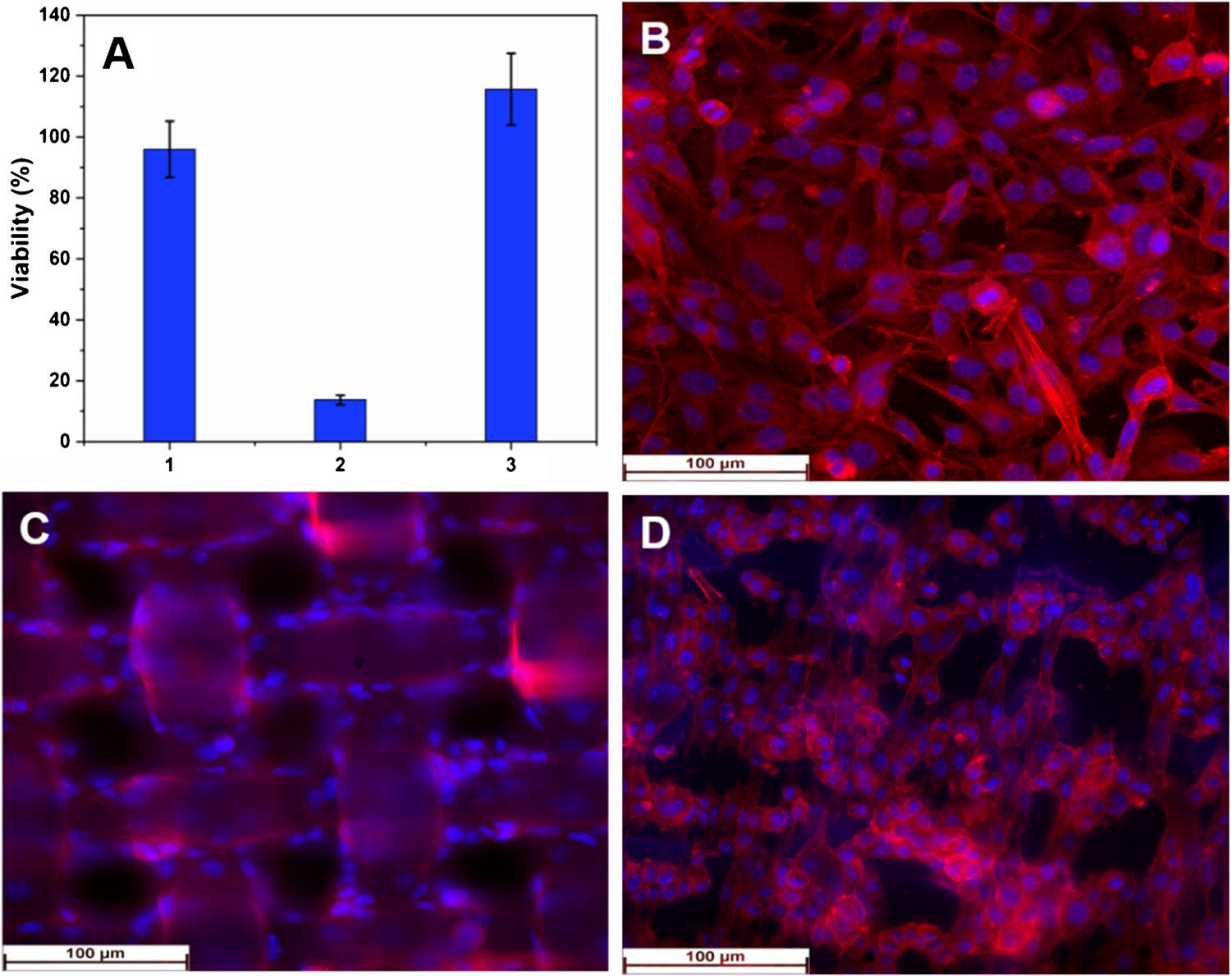
**A** Viability assay and fluorescence images of B16 melanoma cells seeded: (1)** B** on the control, (2) **C** commercial adhesive polymeric membrane, and (3)** D** electrospun fibrillary platform; Phalloidin-iFluor™ 647 was used as dye for staining the cytoskeletal structures and DAPI for nuclei

In this context, the proposed fibrillary scaffold removes the disadvantages of using conventional detection techniques mentioned above in several ways. The commercial membrane used as a base for the proposed scaffold is specially designed to be used with planar screen-printed electrodes and is having the pore size of about 625 µm^2^. These membranes allow the use of small volume of sample while maintains the same electroactive surface of the working electrode. However, the large size of the membrane pores allows the growing of a small number of cells and consequently a low number of biomarkers molecules related to the membrane area. The modification of the membrane with the biocompatible electrospun fibers will result in a high increase of cell number (Scheme 1 and Figs. 2 and 3).

Furthermore, if the cells were cultivated directly on the electrode surface, the electroactive surface decreased for at least two considerations: (1) the adherent cells are blocking the electrode surface meaning that at 70% confluence only 30% of electrode surface remains available for biomarker detection, and (2) the cultivation of cells at the electrode surface request at least 24-h incubation of surface in culture medium and during this time the surface can be passivated by the adsorption of small molecules from medium. Nevertheless, the cells in direct contact with the working electrode may be damaged by the flow charge during the measurement (Fig. 1).

Moreover, the use of electrochemical detection for identifying in vitro cell biomarkers presents some benefits (fast, low cost, handy, and easily accessible method) comparing with spectroscopic or fluorescent techniques, the most important one being that it allows the development of real time monitoring (bio)sensors.

### Electrospun fibrillary scaffold characterization

A novel scaffold configuration for in vitro electrochemical detection of almost any kind of biomarkers released by cells in culture medium was developed by combining electrospun nanofibers obtained through electrospinning and commercial polymeric adhesive membranes.

Beyond the mentioned shortcomings, the commercial polymeric membrane offers the advantage of providing mechanical stability and easy of handling of the scaffold and it acts as a proper support to successfully electrospun the nanofibers on its surface at a proper density in order to offer a suitable environment for cell development. In this way, the cells can be seeded, grown and treated on the proposed scaffold independently of the electrode, avoiding electrode contamination and/or passivation or cell damage improving in this way the longevity and accuracy of the measurements.

The obtained scaffolds were optical and morphological characterized after each preparation step. In Figure S1, a digital photo of the native commercial adhesive polymeric membrane (Figure S1**A**) and its optical microscopy image (Figure S1**B)**, electrospun nylon 6/6 nanofibers scaffold (Figure S1**C),** and B16 cells seeded scaffold (Figure S1**D)** are shown. It can be observed that the large pores of the polymeric membrane did not support the cell adhesion and proliferation. Once the membrane has been covered with nanofibers, the pores become narrow and along with poly-L-Lysine coating the cellular adhesion improves, facts demonstrated also by fluorescence microscopy images.

Morphological analyze shows the regular shape of the polymeric membrane with pores of about 55 μm and fiber diameter approximatively 20 μm (Fig. 2A, A’). After the electrospinning process, the nanofibers uniformly cover the membrane surface and the average fiber diameter is evaluated to be about 120 nm (Fig. 2B, B’). It is obvious that the fiber density is an essential parameter that can be adjusted by changing the fiber collecting time. A low fiber density will not improve the membrane features. By covering the polymeric membrane with nylon 6/6 meshes with optimum density, the B16 cells were able to perfectly accommodate, to remain viable and proliferate on the surface of the resulted scaffold (Fig. 2C, C’). The cells uniformly spread on the platform surface and presented a normal appearance, the presence of the nanofibers preventing the cells from sliding through the pores of the membrane.

The biocompatibility of the designed scaffold and its efficiency in supporting and promoting cell growth and proliferation were assets through MTS viability test (Fig. 3A) and fluorescence images analysis (Fig. 3B–D). According to the viability assay, the B16 melanoma cells seeded on commercial adhesive polymeric membranes were low, in contrast to the high viability of cells seeded on the electrospun fibrillary scaffolds, which was similar to that of the control.

The results of the MTS viability assay were supported by fluorescent imaging analysis, which showed that the viability and proliferation of cells on electrospun fibrillary platforms were similar to that of the control and significantly higher than that of cells on non-electrospun membranes, which have a structure with large spaces through which cells can pass through. Additionally, the cells on electrospun fibrillary platforms appeared to align in accordance with the orientation of the nanofibers.

Same results were obtained by seeding the scaffolds with L929 cells; namely, they adhere and proliferate by having a normal behavior which demonstrate the idea that the scaffolds can be employed for developing a variety of (bio)sensors.

### Figures of Merit–melanin detection

One of the natural melanin synthesis processes in mammalian organisms can be triggered by UV radiation on melanocytes in a process that begins with the activation of melanocyte-stimulating hormone (MSH) receptors on the surface of melanocytes. Inside melanocytes, an enzyme called tyrosinase is activated in response to MSH binding to its receptors. Tyrosinase is a key enzyme in the melanogenesis pathway, as it catalyzes the conversion of the amino acid tyrosine into dopaquinone. Dopaquinone is further converted into various forms of melanin, including eumelanin (responsible for brown and black colors) and pheomelanin (responsible for red and yellow colors). This synthesis pathway involves multiple enzymatic reactions leading to the melanocyte including transcription factor (MITF) which plays a crucial role in regulating melanogenesis.

MITF is responsible for activating the expression of genes involved in melanin production and melanocyte differentiation. MITF levels increase in response to UV radiation and other factors, and it binds to the promoters of genes involved in melanin synthesis, such as tyrosinase and tyrosinase-related protein 1 (TRP-1).

**Scheme 2:**
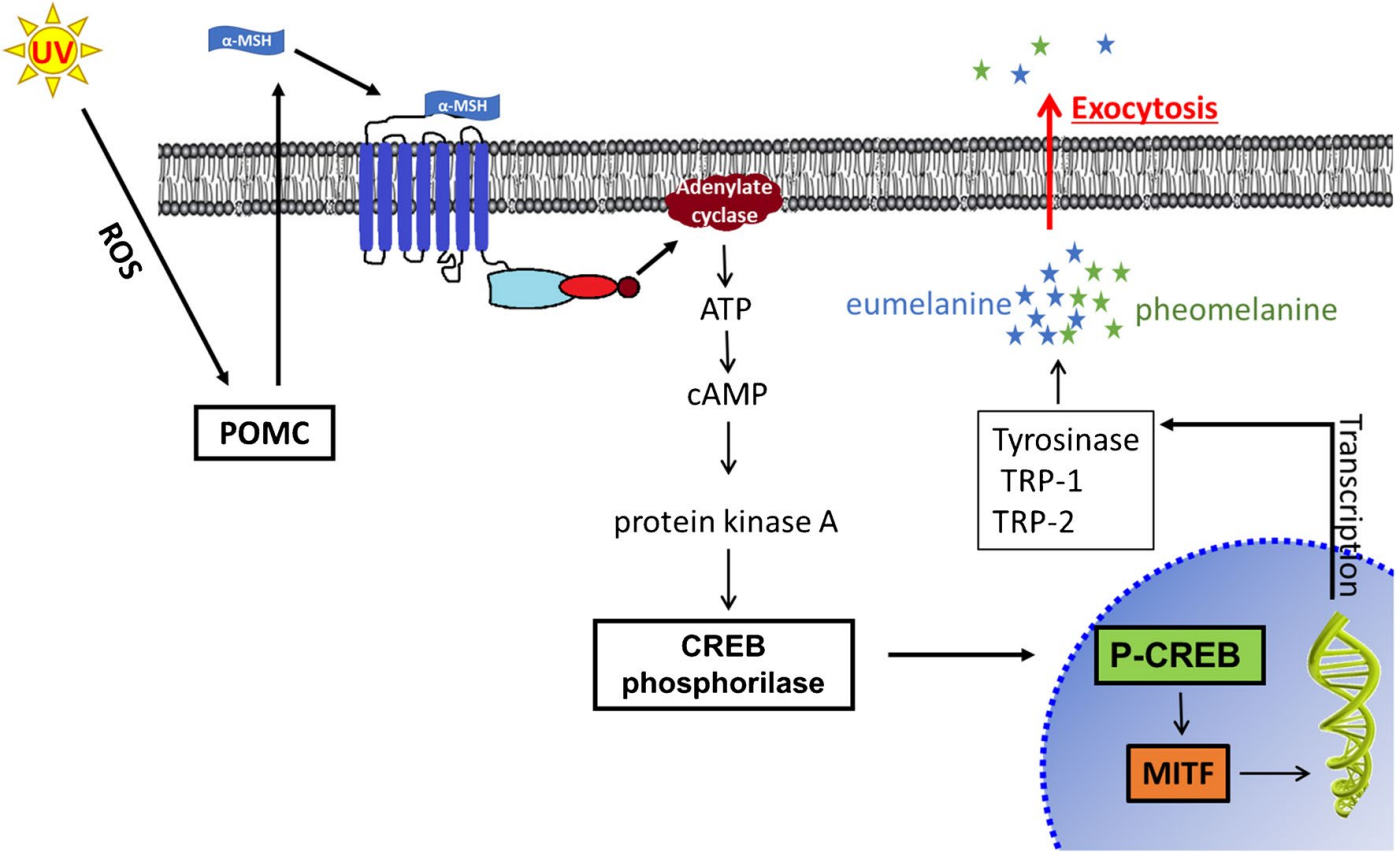
The formation of various forms of melanin when melanocytes are expose to UV irradiation including the MITF generation which is important in regulating melanogenesis

MITF also regulates the transport of melanosomes (specialized organelles for melanin synthesis) from melanocytes to keratinocytes, which are the predominant cell type in the epidermis. As melanin is transferred to neighboring keratinocytes, it accumulates to form pigmentation in the skin, hair, and eyes, providing protection against UV-induced DNA damage and sunburn, according to Scheme 2 [25–27].

In order to demonstrate the working principle of the cell biomarker detection scaffolds, the real-time detection and quantification of melanin produced by the B16 melanocytes during the UV irradiation were achieved using screen-printed carbon (SPC) commercial three-electrode planar transductors consisting of carbon as working electrode, platinum as counter and silver as reference.

The use of presented scaffold and methodology allow the electrochemical detection to be temporarily correlated with the exocytosis release of cells upon certain stimulus. Either intern or extern the stimulus will induce a cascade mechanism that finally will result in an extracellular release that can be measured in an appropriate experimental setup.

**Fig. 4:**
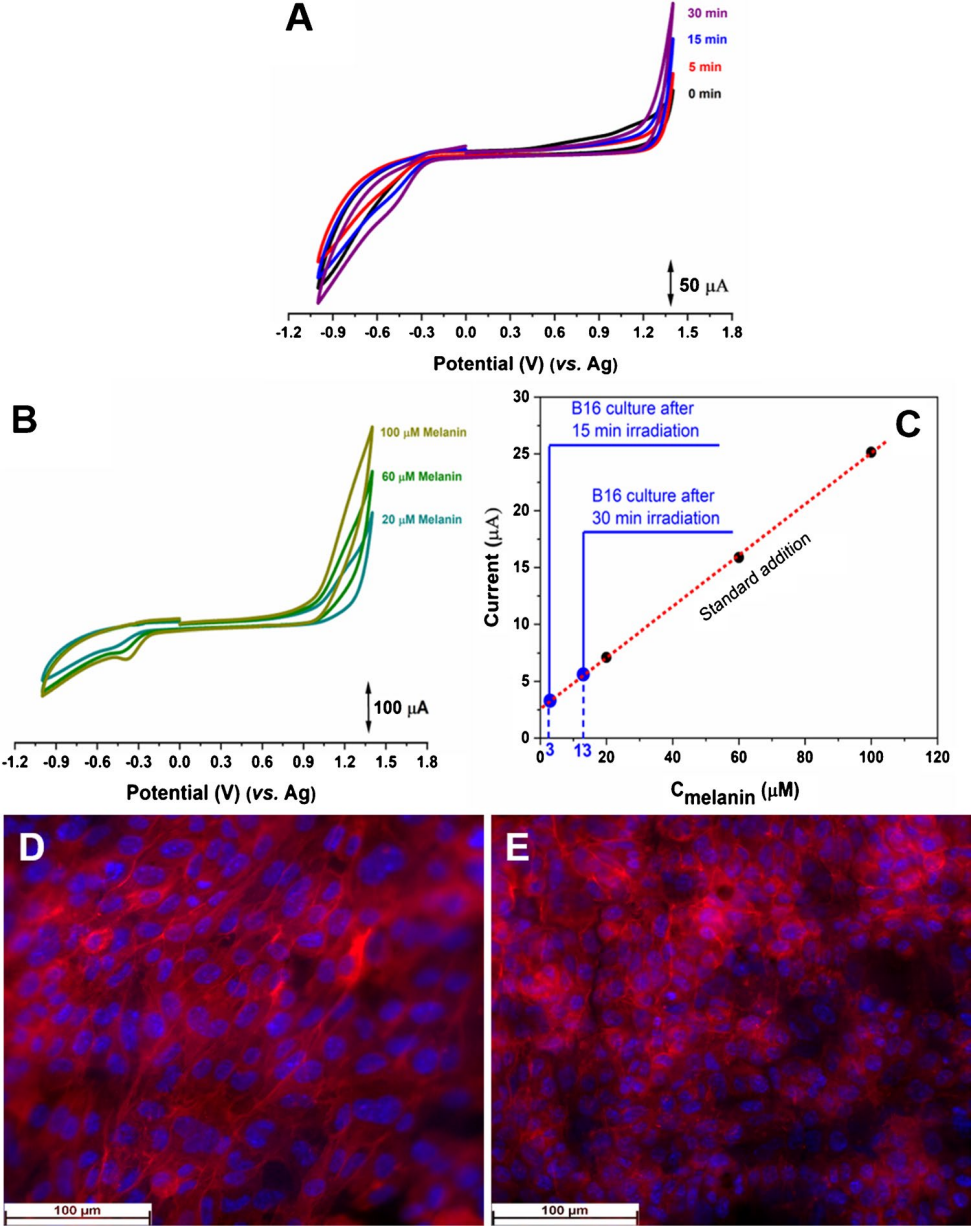
**A** Cyclic voltammograms of melanin detection at corresponding irradiation times; **B** CVs recorded when different melanin solution concentrations were added; **C** registered current as function of melanin concentration for 15- and 30-min irradiation times; Fluorescence microscopy images of the fibrillary platform seeded with B16 melanoma cells **D** before and **E** after 30-min irradiation; Phalloidin-iFluor™ 647 was used as dye for staining the cytoskeletal structures and DAPI for nuclei

Electrochemical detection of melanin was conducted in both anodic and cathodic regions, with a focus on the reduction peak at approximately − 0.4 V (Fig. 4A). The cell culture underwent irradiation for 5, 15, and 30 min, and after each irradiation session, a cyclic voltammogram was recorded. To quantify the melanin produced by cells during irradiation, various concentrations of melanin (20, 60, and 100 µM) were introduced and measured. On the positive going scan, no oxidation reaction appeared, only an increase of the current, regardless of the irradiation time. Previously, such behavior, at boron dropped electrode was associated with cellular release of molecules during a chemical stimulus [28]. However, reversing the scan direction, a cathodic peak appeared at *E*_*p*_ =  − 0.4 V, and the reduction current rises by increasing the irradiation time, as can be seen in Fig. 4A.

After several irradiations, the assembly was carefully rinsed with PBS, a fresh culture medium was added, and several voltammograms were recorded after addition of melanin as standard, at 20, 60, and 100 µM (Fig. 4B). On the calibration plot for standard addition presented in Fig. 4C, it was found a concentration of 3 and 13 µM melanin after irradiation for 15 and 30 min, respectively. These values are in the range of extracellular melanin concentration reported after exposure of the cell culture to different chemical stimulus [13].

The florescence microscopy images presented in Fig. 4D, E recorded before and after irradiation showed no significant differences on the cell morphology and viability demonstrating that the irradiation at 395 nm only induced the release of melanin and had no influence on the cell integrity.

## Conclusion

The preparation steps of the electrospun fibrillary scaffold employed for biomarker detection were successfully adjusted in order to remove the issues encountered when classical detection techniques are employed. Optimum electrospun networks were combined with commercial adhesive polymeric membranes for developing versatile biosensing platforms which support the cell adhesion, growth and proliferation, ensuring a proper environment for electrochemical detection of almost any kind of biological molecule released by cells in culture medium. The scaffolds were optical and morphological analyzed, and their biocompatibility was evaluated utilizing the melanoma B16 and fibroblast L929 cells. The utility of the prepared scaffolds was demonstrated by safely transposing the ensemble B16 cells/scaffolds on screen-printed carbon electrodes and quantifying the melanin production under UV stimulation. It is worth mentioning that the in vitro quantification of melanin released by cells in culture medium requires the use of spectroscopic or fluorescent techniques and laboriously protocols. Through this methodology, the nanofibers based scaffold effectively augment the available surface area for cell growth, minimizing the risk of the electrode passivation, and remove the necessity of using high volume of analytes. These results will stand up the development of point-of-care devices capable to provide real-time information about different kind of complex cellular events or non-invasive monitoring physical, chemical, or biological parameters.

### Supplementary Information

Below is the link to the electronic supplementary material.

Supplementary file1 (DOCX 667 KB)
