# Supplementary material for: Electrospun fibrillary scaffold for electrochemical cell biomarkers detection
Source: Mikrochim Acta. 2024 Jun 29;191(7):435. doi: 10.1007/s00604-024-06523-w (PMC11217050; doi:10.1007/s00604-024-06523-w)
Supplement: Supplementary file 1 — Supplementary file1 (DOCX 667 KB) [file 604_2024_6523_MOESM1_ESM.docx]

# Supplementary Information

# Electrospun fibrillary scaffold for electrochemical cell biomarkers detection

## Mihaela Beregoi^1,#^, Daniela Oprea^1,2,#^, Mihaela Cristina Bunea^1^, Monica Enculescu^1^, Teodor Adrian Enache^1,*^

^1^National Institute of Materials Physics, Functional Nanostructures Laboratory, Atomistilor Str. 405A, 077125, Magurele, Romania.

^2^Faculty of Physics, University of Bucharest, Atomistilor Str. 405, 077125, Măgurele, Romania

^*^corresponding author: [adrian.enache@infim.ro](mailto:adrian.enache@infim.ro)

^#^these authors contributed equally


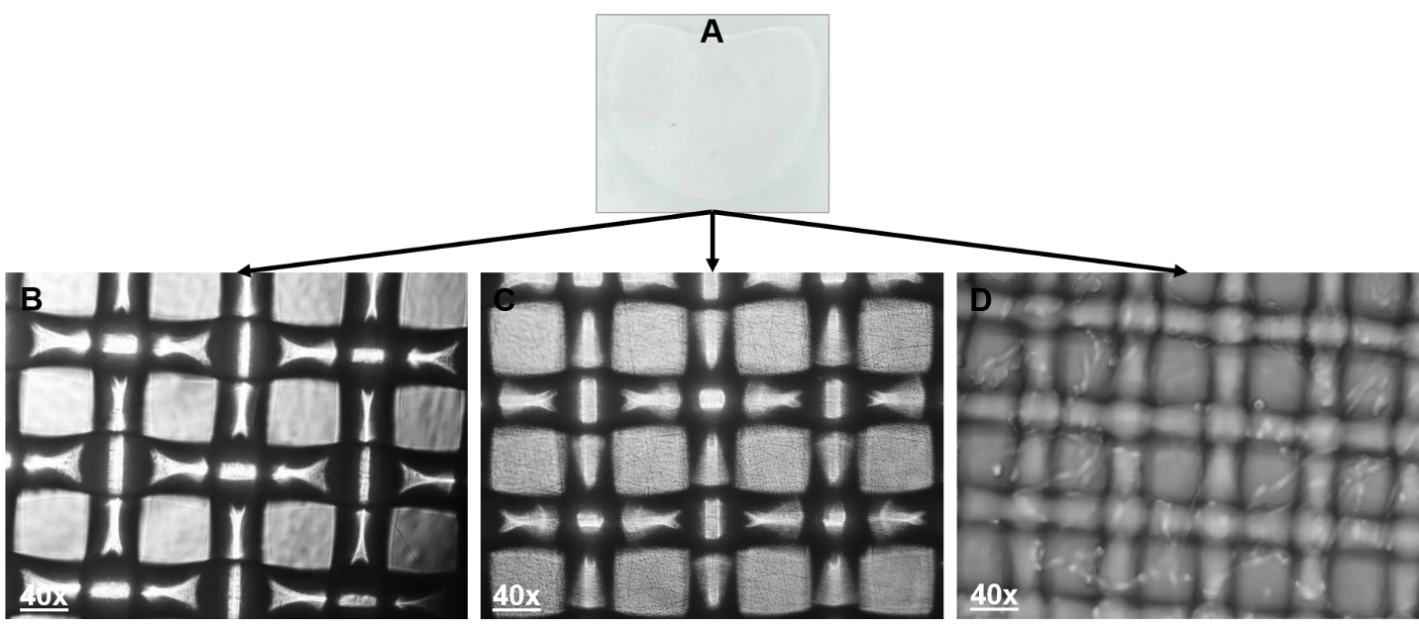


**Figure S1.** (A) Digital photo of the commercial adhesive polymeric membrane and its (B) optical microscopy image; Optical images of the electrospun fibrillary platform (C) without and (D) with B16 melanoma cells.
